# Biobanked human foreskin epithelial cell sheets reduce inflammation and promote wound healing in a nude mouse model

**DOI:** 10.1186/s12896-021-00672-z

**Published:** 2021-02-02

**Authors:** Dongliang Zhang, Jialiang Shao, Jingming Zhuang, Shukui Zhou, Shuo Yin, Fuyue Wu, Jiangang Hou, Xiang Wang

**Affiliations:** 1 Department of Urology, Shanghai General Hospital, Shanghai Jiao Tong University School of Medicine, Shanghai, China; 2 Department of Urology, Huashan Hospital, FuDan University, Shanghai, China; 3 Department of Urology, Sichuan Cancer Hospital & Institute, Sichuan Cancer Center, School of Medicine, University of Electronic Science and Technology of China, Chengdu, China; 4 Remed Regenerative Medicine Clinical Application Institute, Shanghai, China

**Keywords:** Epithelial cell sheets, Human foreskin, Programmed cryopreservation, Refrigerated storage, Biobanking, Fructose, Inflammation, Wound healing

## Abstract

**Background:**

Human epithelial cell sheets (ECSs) are used to clinically treat epithelial conditions such as burns, corneal blindness, middle ear cholesteatoma and vitiligo. As a widely used material in clinic, there is little information on the biobanking of ECSs and its repair effect after storage.

**Results:**

Two methods for biobanking foreskin ECSs were compared in a short term (7 days): 4-degree storage and programmed cryopreservation. Cell sheet integrity, viability, apoptosis, immunogenicity, mechanical properties and function were evaluated. In vivo, ECSs were directly transplanted to skin defect models and histological examination was performed at 1 week postoperatively. We successfully extracted human foreskin-derived primary epithelial cells and fabricated them into ECSs. Compared with 4-degree storage, programmed cryopreservation preserved the ECS structural integrity, enhanced the mechanical properties, decreased HLA-I expression, and increased cell viability and survival. An increased proportion of melanocytes with proliferative capacity remained in the cryopreserved sheets, and the undifferentiated epithelial cells were comparable to those of the fresh sheets. In vivo, cryopreserved ECSs could reduce inflammatory cell infiltration and promote connective tissue remodeling, epithelial cell proliferation and vascular regeneration.

**Conclusions:**

Programmed cryopreservation of ECSs was superior and more feasible than 4-degree storage and the cryopreserved ECSs achieved satisfying skin wound healing in vivo. We anticipate that the off-the-shelf ECSs could be quickly used, such as, to repair human epithelial defect in future.

**Graphical abstract:**

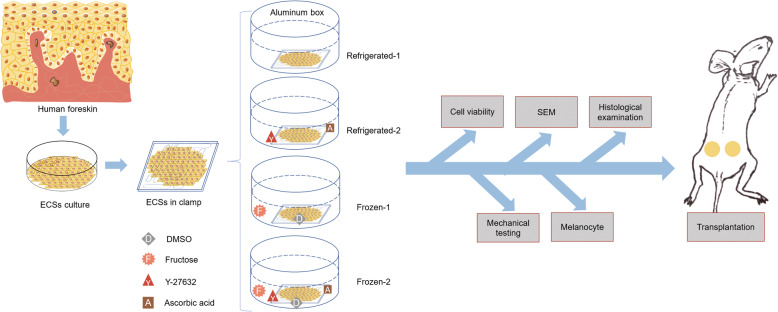

## Background

Epithelial cell sheets (ECSs), as a scaffold-less material, are widely used to repair various epithelial tissue defects, including those in the skin [1, 2], cornea [3], middle ear mucosa [4], urethra and bladder mucosa [5, 6]. Many advantages of ECSs in transplantation are highly beneficial and include the densely arranged epithelial cells that can completely cover the wound surface and the well-preserved extracellular microenvironment that can reduce apoptosis. However, even though ESCs seem to be promising for clinical epithelial cell regenerative therapy, the engineering of cell sheets is time consuming, with several weeks required to generate a transplantable graft. In this process, the tissue was first obtained from the donor, and the primary cells were extracted. After 2 weeks, the cell sheets are generally ready to be harvested and transported from the production center to the surgery center for transplantation. In some cases, surgery would be delayed due to various unexpected changes in the scheduled date of transplantation. If the prepared cell sheets cannot be banked, great amounts of time and financial resources are wasted. Therefore, it is necessary to stock enough engineered cell sheets to ensure that a supply is available to deal with unpredictable factors [7]. This would require an efficient preservation technique that can store engineered grafts for several weeks or months and be ready for use immediately. At present, 4 °C is the commonly used storage temperature during transportation, which can maintain most cell vitality within a few hours after harvest. However, the cell viability would decrease rapidly over time that ECSs couldn’t meet the quality control requirements of transplantation.

Programmed cryopreservation usually includes phase cooling and liquid nitrogen storage and is widely used for organ, tissue, and cell preservation. It has the advantages of ready access, long-term storage, and convenient transport. As many studies have confirmed, the influence of the freezing time on cell viability is negligible. In our previous studies, short-term cryopreserved ovaries [8, 9] and bladder mucosa [10] survived in the transplanted subjects. Moreover, various tissues, such as cartilage [11] and heart valves [12, 13], have been successfully cryopreserved and banked for more than 6 months and achieved transplantation effects that were equivalent to those of fresh controls. Thus, it seems more feasible to store and transport the cell sheets by cryopreservation techniques.

Although numerous protocols have been developed for cryopreserving different cell sheets [14–17], there is little information on the cryopreservation of ECSs. The ECSs are usually composed of 3–5 layers of cells and extracellular matrix. Its cryopreservation requires the maintenance of cell activity and the cell sheet integrity. This is distinctly different from the cryopreservation of a single-cell suspension or an intact organ. Based on modified cryopreservation techniques of bladder mucosa and ovaries, we evaluated the cell viability, integrity, mechanical properties, immunogenicity and in vivo transplantation after storing ECSs for 1 week.

## Results

### Integrity and surface of the preserved ECSs

**Fig. 1:**
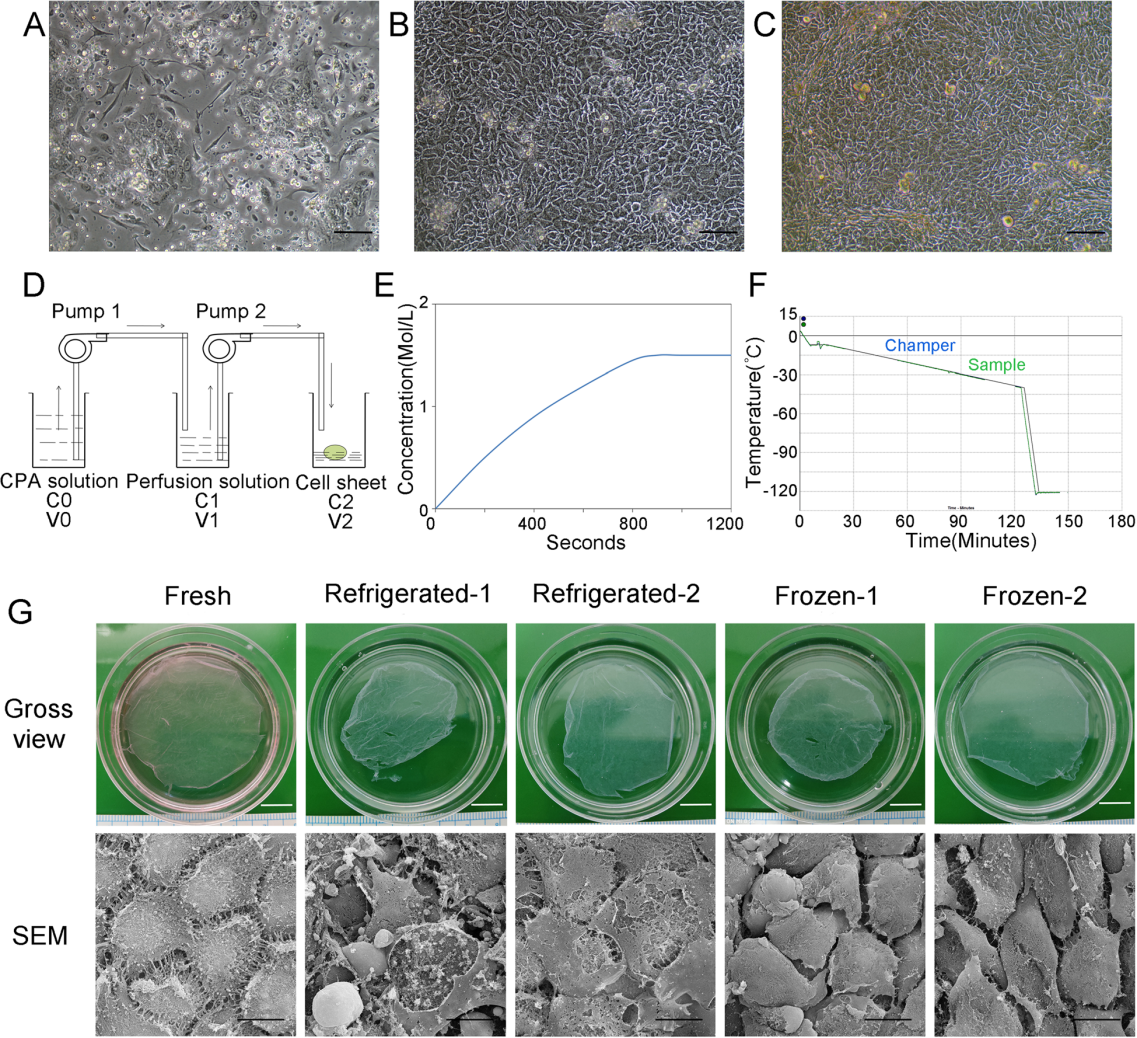
Epithelial cell culture and storage of ECSs. Primary epithelial cells (P0) (**a**) extracted from adult foreskin tissue. P1 epithelial cells show a typical paving stone-like morphology (**b**). Another 7 days, the epithelial cells were more compact (**c**) and continuous ECSs could be harvested. In the two-pump system (**d**), CPAs were pumped into the perfusion solution for mixing and then pumped into the ECS container. The DMSO concentration in the ECS container was slowly increased (**e**) until reaching 1.5 M. Subsequently, the ECSs were transferred to a pre-programmed freezer (**f**) and finally transferred to liquid nitrogen. Compared to the fresh controls, SEM demonstrated that cell membranes and intercellular connections ruptured in refrigerated groups(**g**). The frozen-2 group maintained an intact appearance and preserved most of the cell microstructure. Scar bar: A-C, 50 μm; Gross view, 1 cm; SEM, 20 μm

HFKs were successfully isolated and fabricated into ECSs, and the morphologies of P0, P1 and ECSs under a light microscope are shown in Fig. 1a-c. HFKs originating from foreskin show typical characteristics, including colony formation and a cobblestone-like shape. By the two-pump perfusion system (Fig. 1d), cryoprotectant DMSO was introduced with a linear gradient (Fig. 1e). The cooling program was accurately running as pre-set (Fig. 1f). After storage at 4 degrees for 1 week, ECSs in the refrigerated-1 group showed visible damage, suggesting that ECSs were severely degraded (Fig. 1g). No visible damage was observed on ECSs in the refrigerated-2 group. Cracks of ECSs in the frozen-1 group were observed. The ECSs in the frozen-2 group were similar in appearance to the fresh group, and no obvious structural damage was seen. Compared to those of fresh controls, SEM images of ECSs (Fig. 1g) revealed that the cell membrane and intercellular microstructure were severely damaged after storage at 4 degrees but were well protected in cryopreservation groups.

### Cell viability and apoptosis of the preserved ECSs

**Fig. 2:**
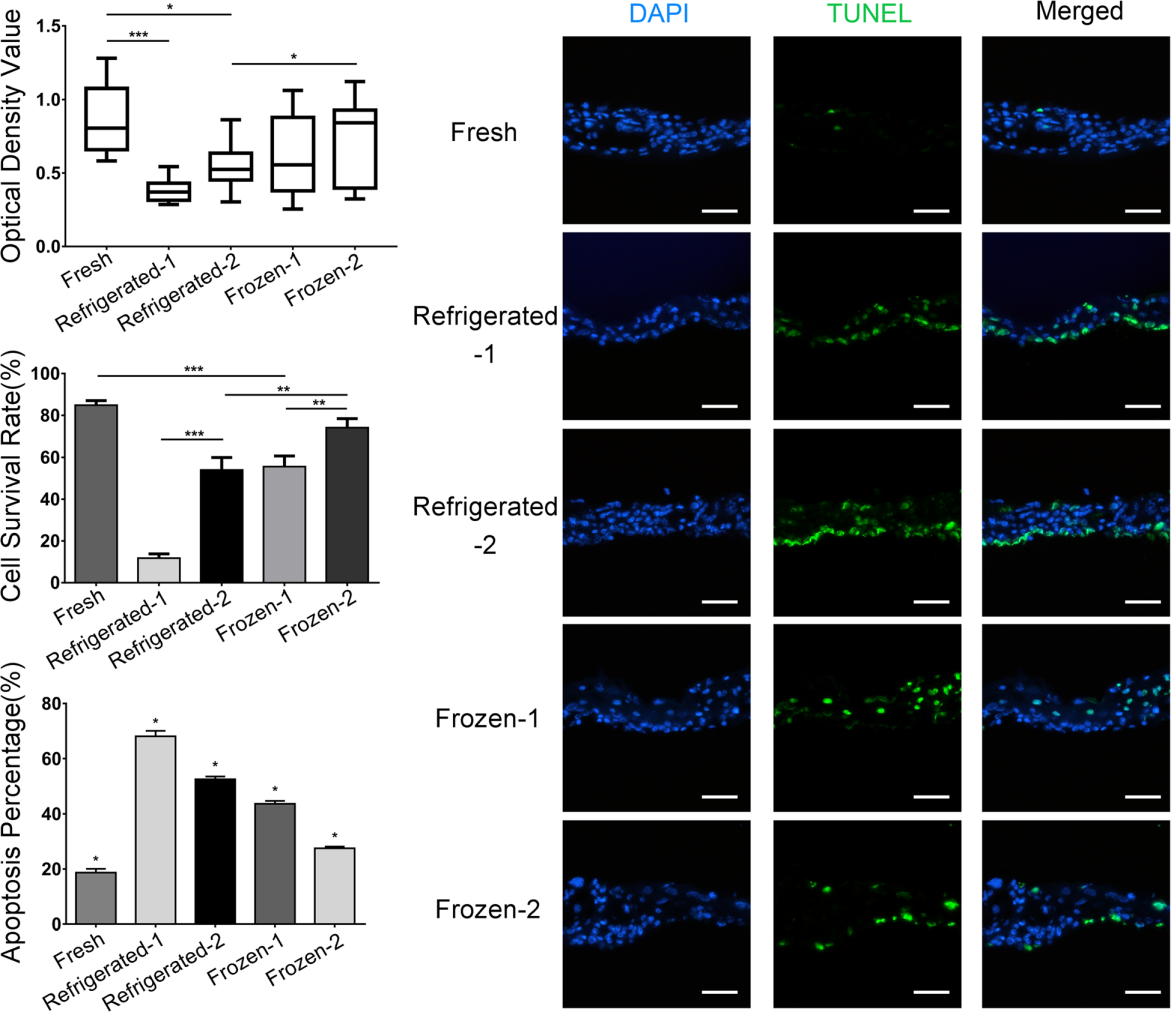
Cell viability and apoptosis of the preserved ECSs. The optical density value showed that the ECS viabilities of the low-temperature storage groups were significantly lower than those of the control group, especially in the refrigerated-1 group. The ECS viabilities of the cryopreserved groups decreased slightly. The cell survival rate of each group calculated by trypan blue staining was consistent with CCK-8. In the apoptosis detection assay, compared with the control group, all preserving groups had an increased rate of apoptosis, and the ECSs in the frozen-2 group performed the best in reducing apoptosis. Scar bar: 50 μm

To analyze the effect of low-temperature storage and cryopreservation on cell viability and apoptosis after preservation, we measured the OD value by CCK-8 testing and detected apoptosis by TUNEL staining (Fig. 2). Compared with the fresh ECSs, cells in the refrigerated group had dramatically decreased viability and a significantly increased apoptosis rate. The apoptosis rate also significantly increased in the frozen groups, but it was superior to those of the low-temperature groups. Notably, the cell viability did not significantly decrease in the frozen groups compared with the fresh controls. In addition, we also calculated the cell survival rate by trypan blue staining. The cell survival rate of preserved ECSs decreased to a certain degree. The lowest cell survival rate was observed in the refrigerated-2 group at less than 20%. Of the two cryopreservation groups, the frozen-2 group had a higher cell survival rate and lower apoptosis rate than the frozen-1 group.

### Mechanical properties of the preserved ECSs

**Fig. 3:**
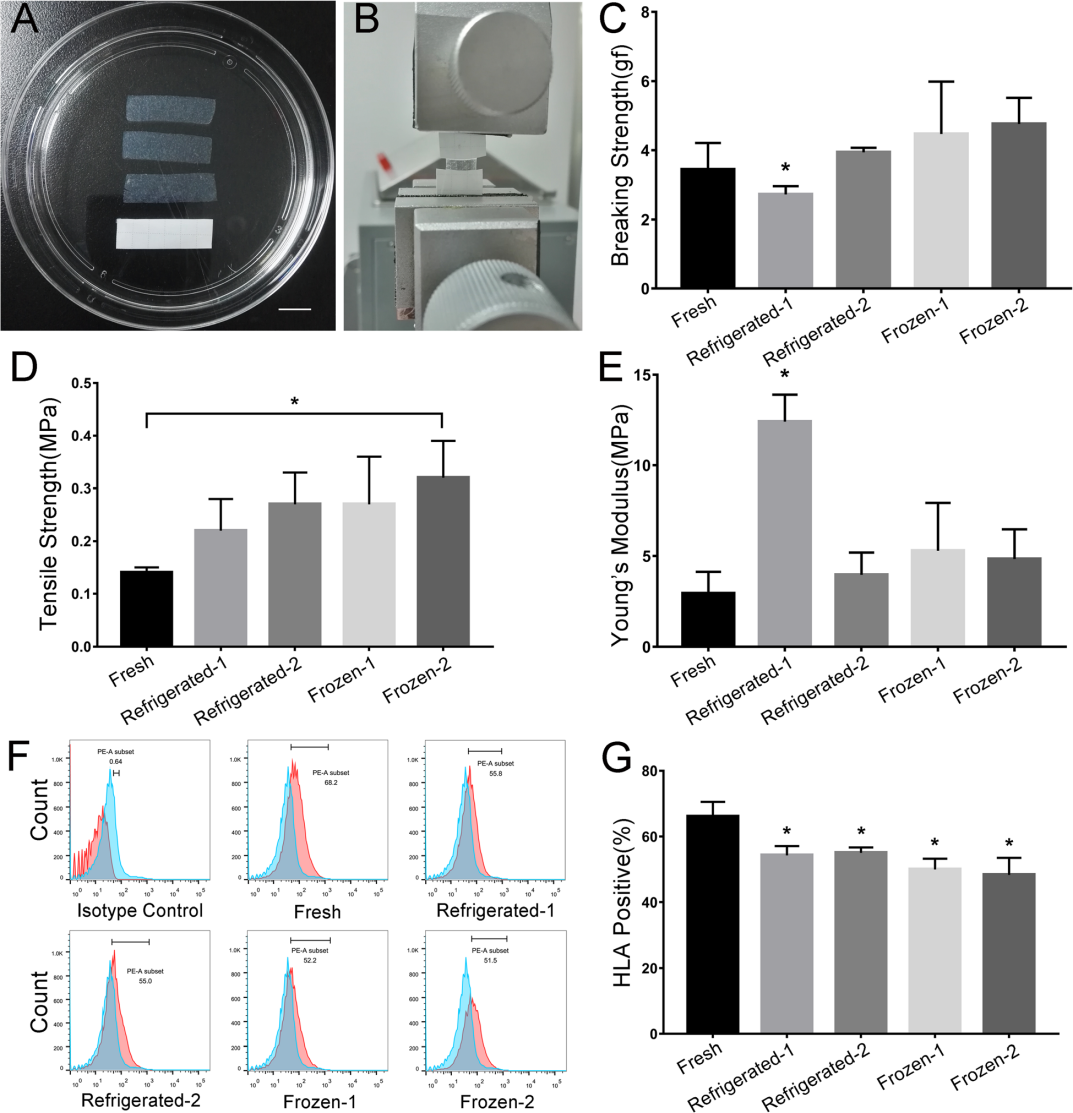
Mechanical properties and immunogenicity of the preserved ECSs. ECSs in each group were cut and tested (**a** and **b**), scar bar: 1 cm. Compared with the control group, the frozen groups were observed to have an increase in the breaking strength and Young’s modulus (**c** and **e**). Compared with the fresh group, the tensile strength in the frozen-2 group was slightly increased (**d**). HLA-I expression in all groups was reduced compared to fresh control group (**f** and **g**)

Passive mechanical testing (Fig. 3a-e) revealed that the breaking strength of ECSs decreased and that Young’s modulus increased significantly in the refrigerated-2 group.

Increases in breaking strength and Young’s modulus in the refrigerated-1, frozen-1 and frozen-2 groups were observed, but the differences were not statistically significant. Compared with the fresh group, a slightly increase of tensile strength was found, but there was no significance, except in the frozen-2 group. Thus, the mechanical properties of the preserved ECSs, except in the refrigerated-2 group, were maintained and possibly enhanced.

### Immunogenicity of the preserved ECSs

The immunogenicity of the preserved ECSs was assessed by flow cytometry analysis of HLA-I. The expression rate of HLA-I, compared to fresh controls (68.4 ± 1.6%), was significantly reduced in both low-temperature storage (refrigerated-1, 56.3 ± 0.6%; refrigerated-2, 55.0 ± 1.7%) and cryopreservation (frozen-1, 52.3 ± 0.9%; frozen-2, 51.3 ± 1.4%) after 7 days (Fig. 3f, g). The results also suggested that the expression rate of HLA-I was not significantly different between the low-temperature storage and cryopreservation groups (*P* > 0.05).

### Melanocyte count in preserved ECSs

**Fig. 4:**
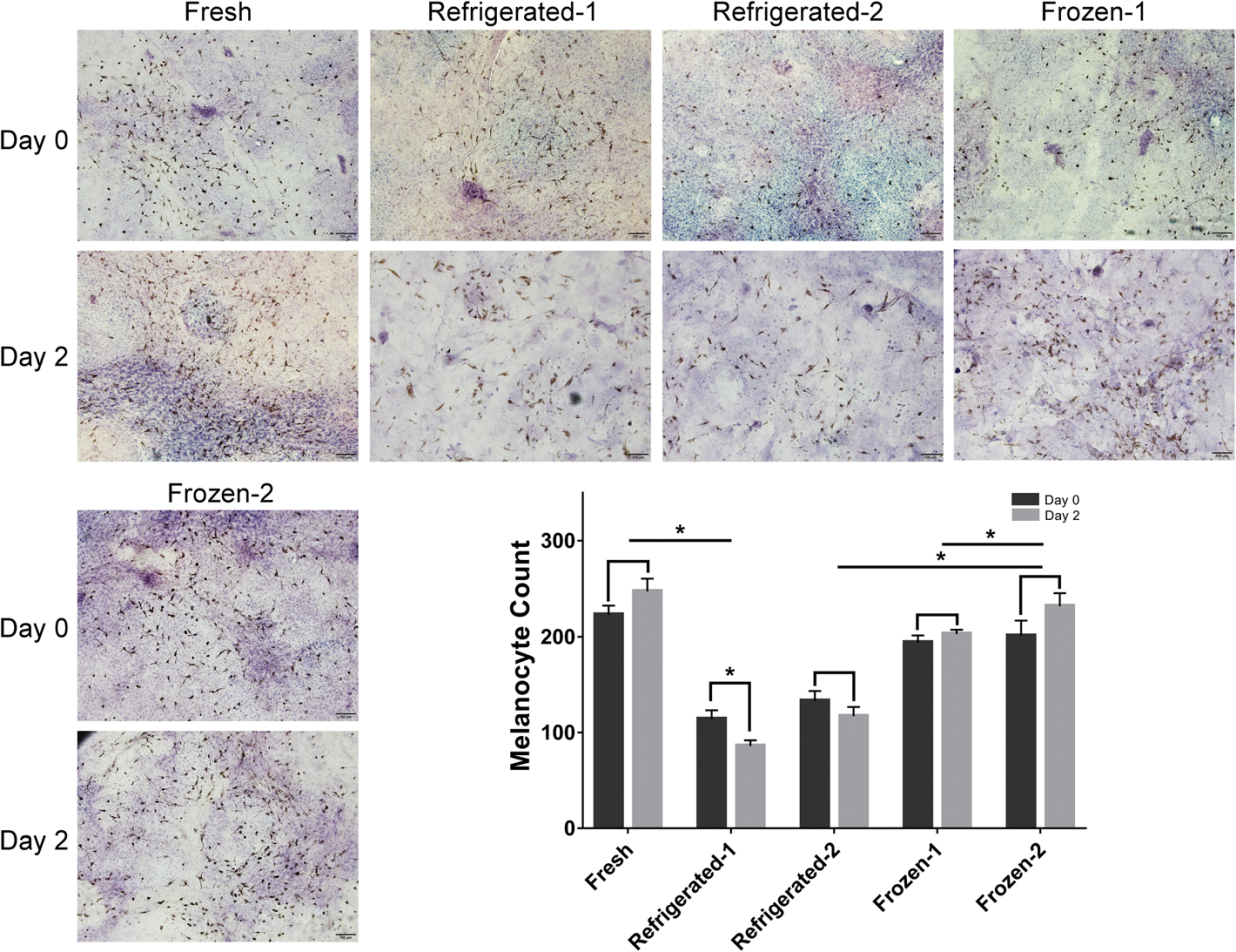
Melanocytes on fresh ECSs and preserved ECSs were counted by DOPA staining. Compared with the fresh control, melanocytes stored at 4 degrees were significantly decreased (*P* < 0.05), and melanocytes further decreased after 2 days of culture. Melanocytes on ECSs in the cryopreservation groups were higher than those in the refrigerated groups (*P* < 0.05). Notably, melanocytes preserved in the frozen-2 group were more abundant than those preserved in the frozen-1 group (*P* < 0.05). Scar bar: 100 μm

The melanocytes in the refrigerated groups stored at 4 degrees were significantly decreased compared with those in the fresh control group (*P* < 0.05) (Fig. 4). The melanocytes further decreased after 2 days of adherent culture, which indicated poor proliferation. Although melanocytes on ECSs also decreased in the frozen groups, cell proliferation was observed after 2 days of adherent culture. The number of melanocytes on ECSs in the frozen groups was higher than that in the refrigerated groups (*P* < 0.05). Melanocytes preserved in the frozen-2 group were more abundant than those preserved in the frozen-1 group (*P* < 0.05).

### Histological and immunohistochemical examination of the preserved ECSs

**Fig. 5:**
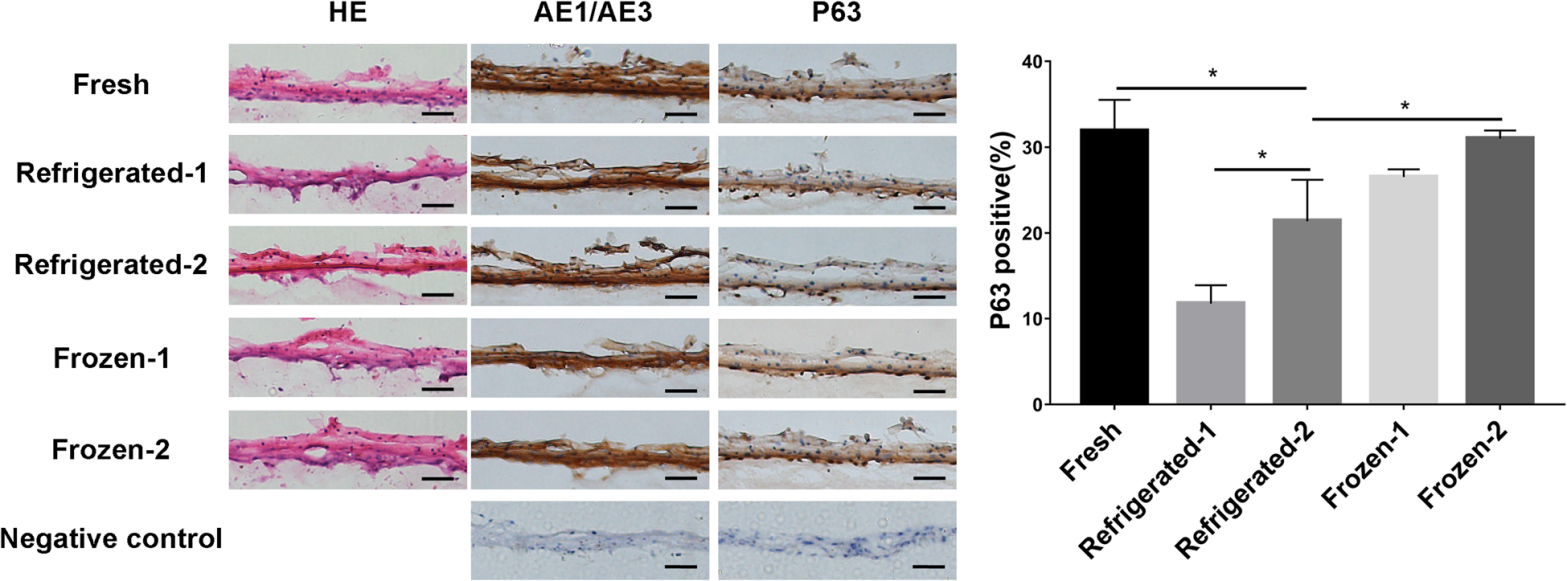
The structure and marker expression of the preserved ECSs examined by HE and immunohistochemistry. Only in the frozen-2 group was the well-preserved structure of ECSs observed with equivalent nucleus density and cell layers to the control group. The expression of AE1/AE3 after storage was similar to that of the control. The percentage of P63-positive cells in the low-temperature groups was lower than that in the fresh control group (*P* < 0.05), and the greatest decrease was observed in the refrigerated-1 group (*P* < 0.05); the greatest preservation was observed in the frozen-2 group (*P* < 0.05). Scar bar: 50 μm

The harvested fresh ECSs were composed of 4–5 layers of basal cells, flattened middle cells, and mature superficial cells (Fig. 5). In the refrigerated-1, frozen-1 and frozen-2 groups, the number of nuclei was sparse, and the cell layers were approximately 2–3 layers. In the frozen-2 group, the nucleus density of ECSs decreased slightly with equivalent cell layers to the control group, and the structure was well preserved. Immunohistochemical analyses revealed that ECSs displayed similar AE1/AE3 expression after storage in each group. As a specific marker for the undifferentiated cell phenotype, P63 was expressed in the basal cells in all groups. The percentage of P63-positive cells in the refrigerated-1 group was lower than that in the fresh control group (*P* < 0.05). A further decrease in the refrigerated-2 group suggested that the positive rate continued to decline over time (*P* < 0.05). The positive cells in the frozen-1 group were comparable to those in the control group (*P* > 0.05). In the frozen-2 group, the percentage of P63-positive cells was higher than that in the frozen-1 group (*P* < 0.05).

### Cryopreserved ECSs facilitate the healing of skin wounds

**Fig. 6:**
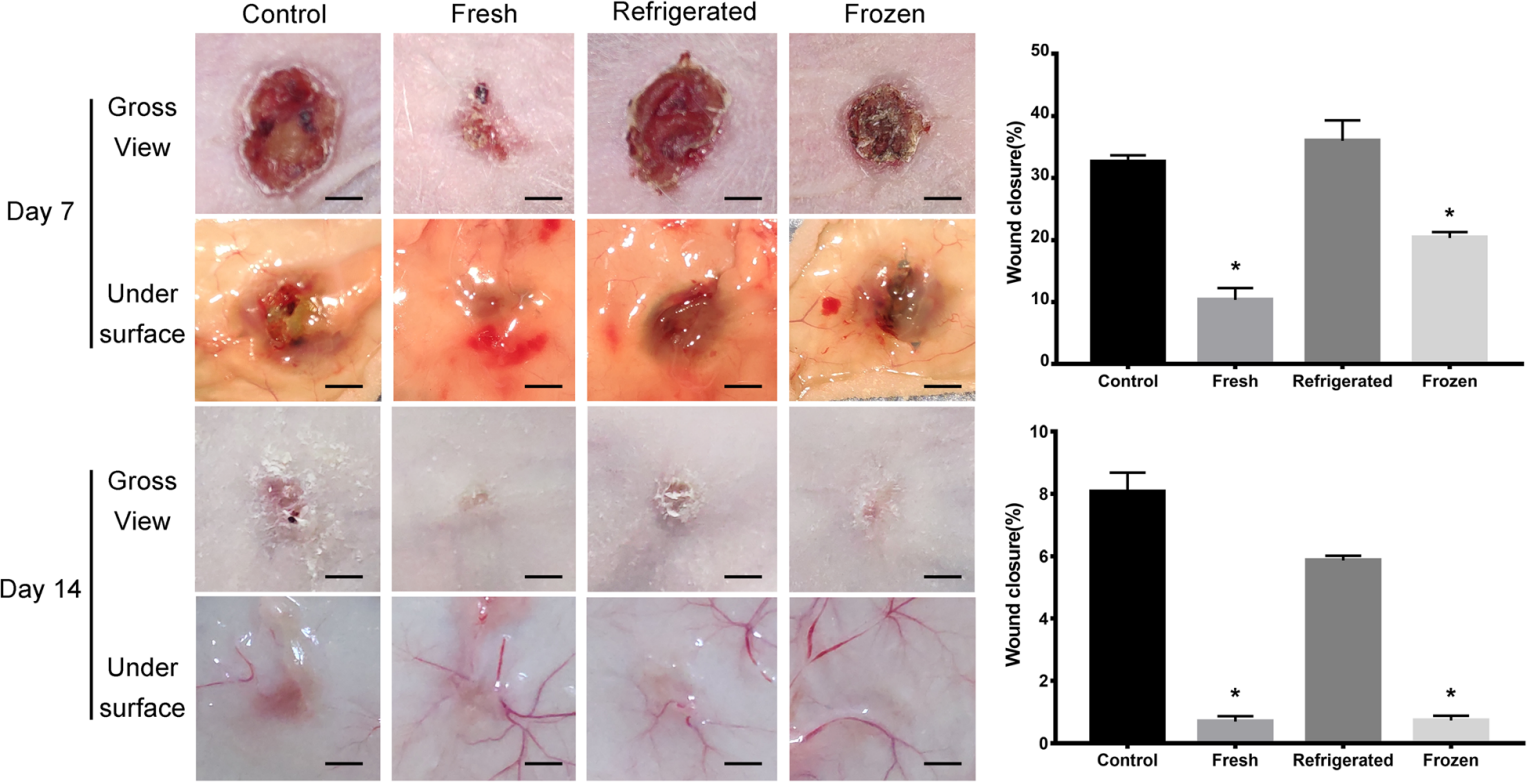
Gross images from the front and undersurface of the skin wounds at day 7 and day 14 postwounding. The wound closure rates were calculated. Scale bar: 2 mm

To further evaluate the stored ECSs in vivo, we used fresh ECSs, refrigerated ECSs and frozen ECSs to repair skin defects in nude mice. As indicated by the representative images of wounds on day 7 and day 14 postwounding and quantification of the wound closure rates, the fresh and cryopreservation groups showed profoundly enhanced wound closure compared with the other two groups (Fig. 6).

**Fig. 7:**
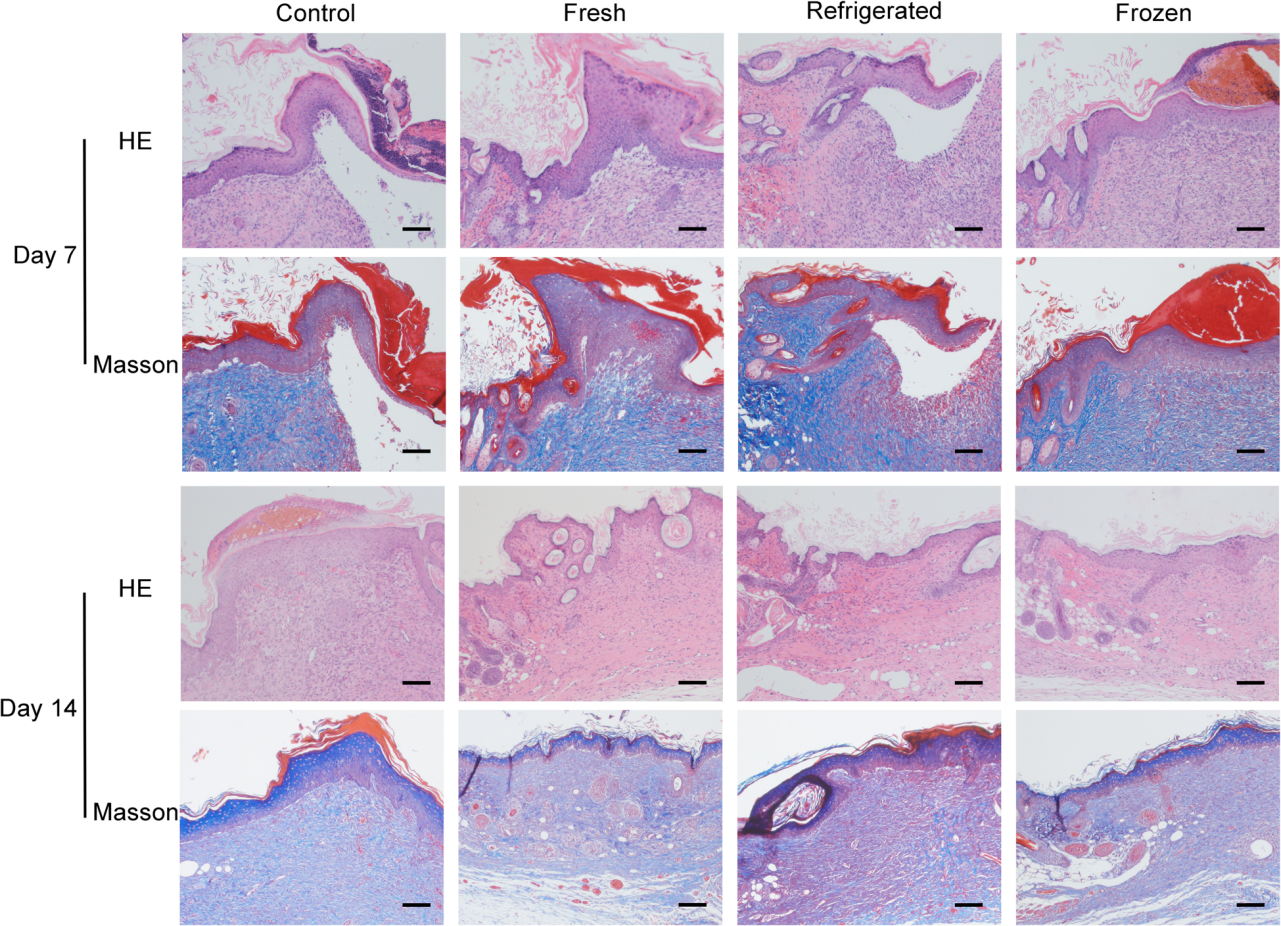
Frozen ECSs facilitated the healing of skin wounds. H&E staining and Masson’s trichrome staining of skin wounds were performed at day 7 and day 14 postwounding. Scale bar: 100 μm

H&E staining showed that the wounds in fresh and cryopreserved groups were nearly closed, whereas large areas of wounds in the other two groups were not covered with newly formed epidermis at day 7 (Fig. 7). As revealed by Masson’s trichrome staining, the wound tissues of the fresh and cryopreservation groups exhibited more regularly and densely arranged collagen fibers than those of the other two groups (Fig. 7). At day 14, we observed intact epithelium, primary skin structure, loose subcutaneous tissue and abundant blood supply in the fresh and cryopreservation groups.

**Fig. 8:**
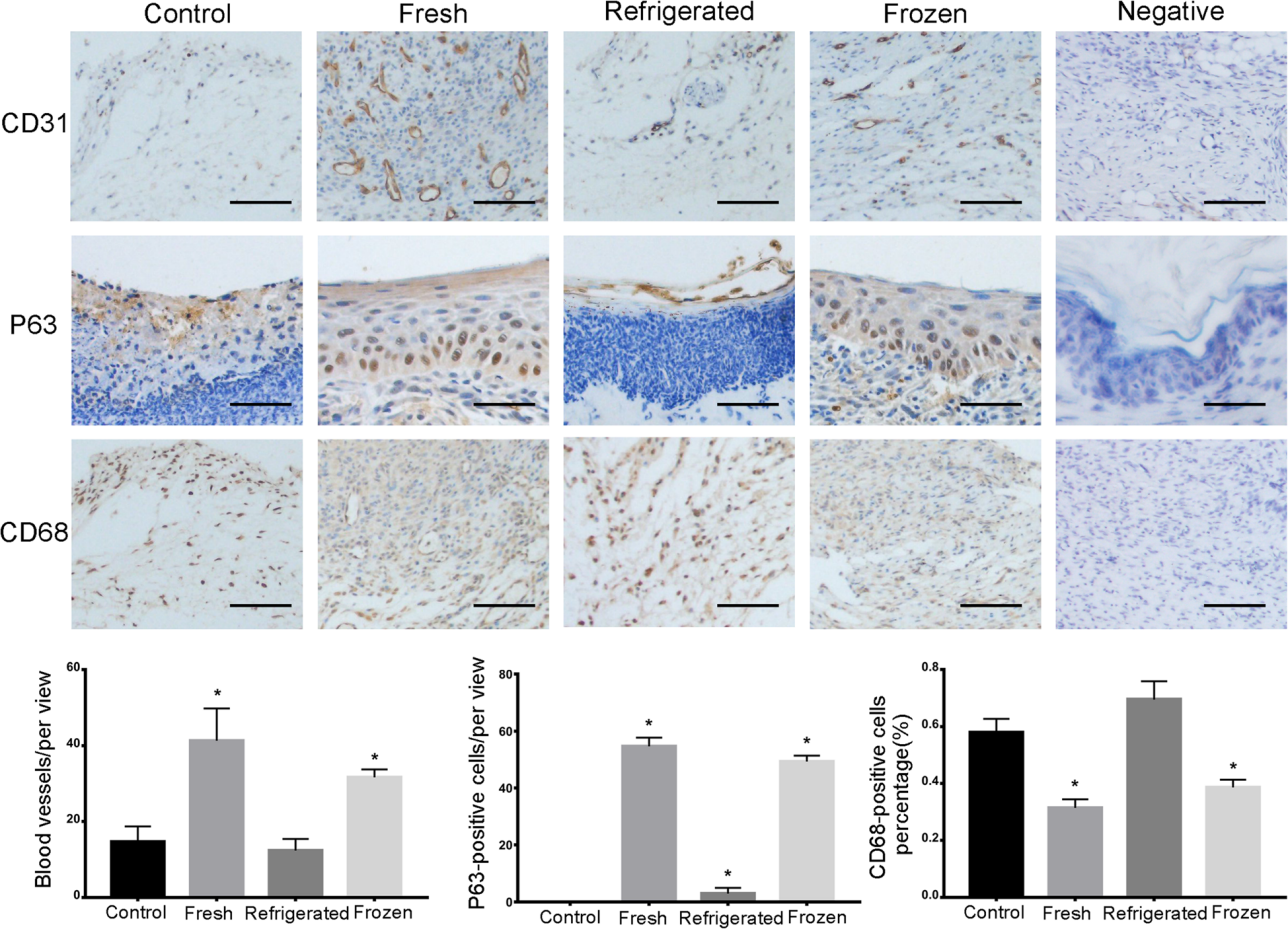
Frozen ECSs promoted basal cell proliferation and increased angiogenesis in the wound areas at day 7. As shown by CD31 staining in wound areas, both fresh and frozen ECS regimens enhanced the formation of blood vessels in the skin wounds. The numbers of P63-positive basal cells indicated many more proliferating epithelial cells in the wound sites of fresh and frozen ECS-treated mice (*P* < 0.05). The percentage of CD68-positive cells showed less infiltration of inflammatory cells (*P* < 0.05). Scale bar: 50 μm

**Fig. 9:**
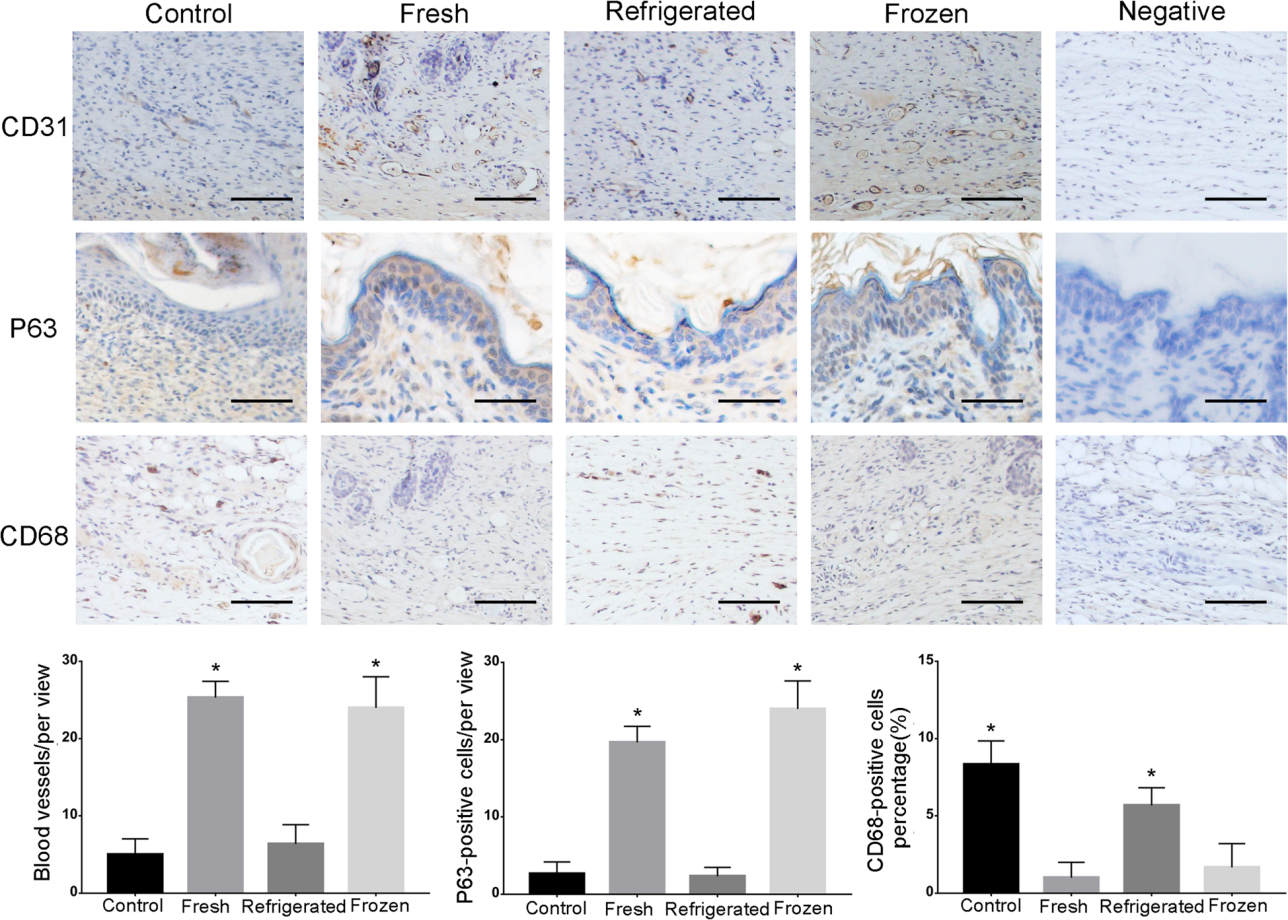
Basal cell proliferation and angiogenesis in the wound areas gradually decreased at day 14 compared to day 7. The density of blood vessels in fresh and frozen groups were higher than control and refrigerated groups. More proliferating epithelial cells in the wound sites of fresh and frozen ECS-treated mice were still found (*P* < 0.05). CD68-positive cells were hardly found in the fresh and frozen groups(*P* < 0.05). Scale bar: 50 μm

P63 staining indicated many more proliferating cells around the wound sites in the fresh and cryopreservation groups compared with the other two groups (Figs. 8 and 9). To determine angiogenesis in the wound sites, we photographed the skin from the undersurface (Fig. 6) and conducted immunochemical staining for CD31 (Figs. 8 and 9). ECSs in both the fresh and cryopreservation groups enhanced the formation of blood vessels in the skin wounds, and the vascular density was much higher than that of the other two groups. Immunohistochemical staining for CD68 revealed less inflammatory cell infiltration in the fresh and cryopreservation groups than in the other two groups (Figs. 8 and 9).

## Discussion

Due to the time-consuming culture procedure of ECSs, there is a pressing need to store shelf-stable ECSs to provide flexibility when planning transplant operations, back-up sheets for reoperations, and longer transportation time. How to maintain the cell viability and phenotype of ECSs in storage or transport is a key issue faced. For the first time, we compared two methods of storing foreskin ECSs in 1 week: 4-degree storage and programmed cryopreservation. We found that the cell viability was significantly improved after adding antioxidant and antiapoptotic components to the preservation solution. Better results were found in programmed cryopreservation in terms of the integral structure, enhanced mechanical properties, high cell viability and cell survival rate. In addition, melanocytes with proliferative ability were also detected on the cryopreserved ECSs. In vivo transformation, the programmed cryopreserved ECSs obtained good repair effect.

After 1 week of storage at 4 degrees, the cell viability of ECSs decreased seriously, and the cell survival rate was less than 20%. Similar to our previous clinical study, it is unfeasible to transfer ECSs stored at low temperatures for more than 1 day [1]. Because it can still metabolize and produce a large number of harmful substances at 4 degrees, resulting in osmotic pressure change and apoptosis. Cell activity would decrease rapidly within a few hours, accompanied by degradation of extracellular matrix (ECM). When the ECM was so seriously degraded as holes were developed, and the mechanical properties were significantly reduced. However, when ascorbic acid and Y-27632 were added to the storage medium, the cell viability, survival rate, nonapoptotic cell rate and mechanical properties of ECSs were improved but inferior to fresh and cryopreserved ECSs. Ascorbic acid is a strong reducing agent that can stabilize cell membranes and reduce oxidative stress damage. Y-27632 inhibits Rho-associated, coiled-coil containing protein kinase (ROCK) by competing with ATP for binding to the catalytic site [18], thereby preventing apoptosis and increasing cell survival. Jackson et al. found the accumulation of mitochondrial reactive oxygen species (ROS) increased differentiation, DNA damage and cell death after storing human ECSs at 4 degrees for 1 week [19]. Although some protective ingredients can be added, the cell activity of ECSs preserved at low temperature is easy to decrease.

More studies have tried to preserve cell sheet by cryopreservation and have successfully cryopreserved limbal stem cell sheets [14], chondrocyte sheets [15], myoblast cell sheets [16], and mesenchymal stem cell sheets [17], in which 48–97% cell viability was reported. However, cryopreservation of ECSs remains a challenge because the ability to tolerate cryopreservation varies by cell type. The unique cell-cell junctions and the less secreted ECM of the epithelial cells can be easily destroyed by ice tension. In view of our previous studies, we continued to use the program-freezing device and two-pump perfusion system. To cryopreserve ECSs, frost-resistant clamps and thermally conductive boxes were used during cryopreservation. Supported by the special clamps, ECSs maintained a flat structure and maintained their integrity during the freezing/thawing process. In our exploratory experiment, due to the random accumulation of ECSs without clamps, ECSs were easily cracked into fragments after thawing (data not shown). Miki Maehara et al. have also attempted to envelope chondrocyte sheets in a thin film, and 86% cell viability was achieved after cryopreservation [20]. In addition, a slightly increase of mechanical properties was found after cryopreservation, but there was no significance. It might be explained that the freezing/thawing process including dehydration of the ECM could increase the crosslinking between collagen fibrils and make the tissues tough and tensile [21].

Cryoprotectant agents played the most important role in the successful cryopreservation of ECSs. To date, there are few data focusing on cryopreserving scaffold-free ECSs, but several studies have reported the viability of keratinocytes seeded on tissue-engineered sheets after freezing/thawing [22, 23]. Fanfan Chen et al. studied the cryopreservation of tissue-engineered epithelial sheets constructed by chitosan hydrogel [22]. The cell viability assay showed that 95% of cells were viable in the non-cryopreserved group, while only 30% were viable in the DMSO group. Pankaj Chopra et al. seeded normal keratinocytes on poly (vinyl alcohol)-carrageenan scaffolds [24]. After cryopreservation for 15 days, the cell viability was determined to be greater than 45%. We attempted to cryopreserve ECSs using classical formulations (10% DMSO), but cell viability was severely decreased (data not shown). After we added fructose into CPA, the survival rate of ECSs was significantly improved more than 50%. As a natural component of nonpermeating cryopreservatives, fructose has been widely used in cryopreservation studies and proven to have excellent protective effects. We also added ascorbic acid and Y-27632 to CPA, because the cryopreservation process could lead to cryo-injuries that disrupt the normal biological function of cells, including osmotic stress, oxidative stress and induction of apoptosis [25]. Expected results were achieved by optimized CPA consist of these cryoprotectants.

We did more tests of the ECS to fully understand the effects of cryopreservation on other aspects. For skin grafts, a certain number of surviving melanocytes is also required to maintain the skin color of the transplant area consistent with the surrounding area [1]. When the epithelial cells were extracted, a small number of melanocytes were usually mixed with. By adding necessary growth factors, the co-survival of melanocytes and epithelial cells could be achieved. Tyrosinase in melanocytes synthesized exogenous levodopa into pigment granules, which made the cells black under microscope. We carried out secondary culture after preservation and found that Melanocytes on ECSs could proliferate after the freezing/thawing process. In addition, we also found that cryopreservation could significantly reduce the immunogenicity of the ECSs, similar to cryopreserved ovaries [9] and bladder mucosa [10] in our previous studies. As the positive rate of HLA-I dropped by only 16%, allogeneic transplantation of ECSs by cryopreservation or low-temperature storage to avoid an immunological reaction remains a challenge.

Finally, we compared the repair effect of ECSs after low-temperature storage and cryopreservation in a skin wound model. It’s showed that frozen ECSs could reduce the infiltration of CD68-positive inflammatory cells and the collagen deposition. CD68-positive cells were mostly expressed in macrophages and a large amount of macrophages infiltration in the early stage of transplantation would lead to collagen deposition and scar formation. Many studies have shown that reducing macrophage infiltration or regulating macrophage polarization can reduce inflammatory response and promote tissue repair [26]. On the other hand, we detected more P63 positive cells in the cryopreserved ECSs, which was conducive to epithelial regeneration. Generally, the undifferentiated cells expressing p63 in the basal layer of the skin are self-renewing and play a vital role in the development and differentiation of epithelial tissue [27]. Retention of the p63-positive cell phenotype in cultured and stored ECSs can improve the success rate of transplantation in the clinic [19]. During the freezing/thawing process, the differentiated epithelial cells on the surface of ECSs were more likely to die and fall off than undifferentiated epithelial cells, which explained that the proportion of P63-positive cells in the frozen group was slightly higher than that in the fresh control group. The survival cells participated in the regulation of host inflammation and repair process by paracrine, and promoted vascular regeneration until the wound was completely covered by epithelium. An increasing number of studies have shown that the grafted cell sheet is not a direct substitute for the host tissue but activates the regeneration potential of the host tissue through adhesion, guidance and secretion [28]. The completely regenerated epithelial layer, high-positive rate of p63 and high-density angiogenesis confirmed that the programmed cryopreserved ECSs had a repair ability comparable to that of fresh ECSs in vivo.

In this study, cryopreservation and biobanking of ECSs for preserving the intact structure and potential function was proven to be feasible. These banked ECSs will make autologous transplant surgery more flexible. Furthermore, programmed cryopreservation and a two-pump perfusion system combined with optimized cryoprotectants enable automated and mass cryopreservation. We confirmed that cryopreservation of ECSs was better than low-temperature storage for 1 week. For long-term storage, cryopreservation was also believed to be competent. However, cryopreservation in our study did not greatly reduce the immunogenicity of the ECSs, and allogeneic transplantation is still facing the most challenging problem of immune rejection. We only compared the short-term repair effect in vivo, and it might be more convincing if a longer time point was set.

## Conclusion

In summary, we successfully extracted human foreskin-derived primary epithelial cells and fabricated them into ECSs and then used programmed cryopreserved ECSs to repair the skin defects of nude mice. By comparing low-temperature storage with cryopreservation, we thoroughly evaluated the integrity, viability, apoptosis, immunogenicity, mechanical properties and cell function of ECSs. The results show that programmed cryopreservation and storage of ECSs using optimized cryoprotectants was feasible and applicable. Cell sheets stored in liquid nitrogen after programmed cryopreservation could also be able to be transported longer distances, demonstrating that building a complete storage and transportation network is possible in the future.

## Methods

### Epithelial cell isolation

All methods were carried out in accordance with relevant guidelines and regulations. And all experimental protocols were approved by the ethics committee of Shanghai Jiao Tong University affiliated Shanghai general hospital. Human foreskin keratinocytes (HFKs) were isolated from young humans undergoing circumcision. The method was described previously [1]. Briefly, most of the fibrous dermal tissue was first removed, leaving a thin dermis and epidermal layer. Then, the layer was cut into 1 mm × 1 mm cube and incubated in 20 ml 0.05% Trypsin-EDTA (Gibco, USA) with 100 μg/ml penicillin and streptomycin antibiotics (Gibco, USA) at 4 °C overnight. The next day, after trypsin-EDTA was quickly neutralized, the tissue cubes were digested with 0.1% collagenase I (Gibco, USA) at 37 °C for 1 h. Finally, primary HFKs were obtained and suspended in keratinocyte growth medium (KGM) with 10% fetal bovine serum (Sigma, USA). KGM was prepared by mixing equal volumes of DMEM and DMEM with Ham’s F-12 medium (Termo Fisher Scientific, USA). KGM supplement was containing 0.3 μM hydrocortisone (SAXIZON, Japan), 140.0 mU/mL insulin (Sigma, USA), 2.0 nM triiodothyronine (Sigma, USA), 0.2 μM epidermal growth factor (Termo Fisher Scientific, USA), 1.0 nM cholera toxin (Wako PureChemical Industries, Japan), 100 μg/mL streptomycin and 0.25 μg/mL amphotericin B (Gibco, USA). HFKs (5 × 10^4^ cells/cm^2^) were seeded in 60 mm culture dishes in 5 ml complete medium (P0) and cultured at 37 °C with 5% CO2. Second-passage cells (P1) were used for the ECS culture.

### Human ECS culture

Second-passage cell suspensions were diluted and seeded in 60 mm dishes at 2 × 10^4^ cells/cm^2^. ECS cultures were maintained with medium changes every day for 2 weeks. ECSs were then peeled off for preservation experiments.

### ECS preservation

Fresh ECSs, not subjected to storage, served as controls (*n* = 3). After harvesting, each ECS was sealed and randomly selected for storage in KGM at 4 degrees (refrigerated-1, *n* = 3). ECSs were also preserved in KGM supplemented with 0.5 mM ascorbic acid (Sigma, USA) and 10 μM Y27632 (Selleck, USA) at 4 degrees (refrigerated-2, *n* = 3). Following 7 days of storage, new KGM was added to the groups and rewarmed in a 37 °C incubator for 1 h. The ECSs and medium were collected for future analysis.

The programmed cryopreservation was performed as described in our previously published studies [8, 10]. Briefly, by a two-pump perfusion system (2132 MicroPerpex, LKB BROMMA, Sweden), cryoprotectant DMSO (Sigma, USA) was introduced with a linear gradient rising from 0 to 1.5 M. Cryoprotectant agents (CPA0) not containing (Frozen-1) or containing (Frozen-2) 0.1 M fructose, 0.5 mM ascorbic acid, and 10 μM Y27632 in KGM were prepared. The ECSs were cryopreserved in triplicate. The ECS was nipped by a designed polytetrafluoroethylene porous clip and then transferred to an Aluminum freezing box (diameter × height, 59 mm × 17 mm) with 15 ml cryopreservation medium. A 5-slot ampoule cryochamber (Planer KRYO 10 Series III Freezer, UK) was run by the pre-set cooling program. The boxes were finally plunged into liquid nitrogen. After 7 days of cryopreservation, thawing was carried out rapidly by holding in air for 1 minute to boil off any liquid nitrogen and swirling in a water bath at 40 degrees. When completely thawed, the ECSs in the frozen groups were washed using the pumps at the same flow rates as before. Interstitial DMSO in the ECSs was progressively eliminated by pumping medium into the reservoir. The ECSs were replenished with new KGM and rewarmed at 37 degrees. After 1 h of incubation, the ECSs and medium were collected for future analysis.

### Integrity and mechanical testing of ECSs

Gross observations were made to determine the integrity of preserved ECSs. For mechanical testing, the preserved ECSs in groups were prepared as longitudinal strips (30 mm in length, 10 mm in width). The ECS thickness was measured by dial thickness gauges. Before testing, those cell sheets were maintained in serum-free medium at 37 degrees. The mechanical properties of the ECSs were measured using an FR-108C system (FARUI, Inc. Shanghai, China) at a constant speed of 10 mm/min. According to the manufacturer’s software, the ultimate loads were recorded and analyzed. Each group was tested in triplicate.

### Scanning electron microscopy (SEM)

Samples were fixed in 1% osmic acid (Sigma, USA) for 30 min at room temperature and dehydrated by an ascending series of ethanol (from 50 to 100%). After air-drying overnight, samples were coated with gold. SEM (VegaII XMU instrument Tescan, Czech-Republic) was used to observe the morphology of ECSs.

### Cell viability assay

CCK-8 testing and trypan blue staining were employed to investigate the viability of the preserved ECSs. For CCK-8 testing (Dojindo, Japan), the ECSs were punched out with a biopsy punch (8 mm in diameter) and transferred into a 96-well plate with 100 μl CCK-8 solution (10 μl CCK-8 in 90 μl medium) in each well. The plate was then incubated at 37 °C with 5% CO2. After 1 h of incubation, the optical density was read on a spectrophotometer (Cytation Hybrid, BioTek, USA) at 450 nm. After incubated in PBS containing 0.05% trypsin-EDTA at 37 °C for 5–10 min, the ECSs were gently pipetted to single cell suspension. Cell viability was determined by staining viable cells with trypan blue (0.4% solution) (Sigma, USA) staining. Viability (%) = Live cells/Live and Dead cells × 100.

### Flow cytometry analysis of human leukocyte antigen class I

The preserved ECSs were first dissociated using the methods above. The cells were then incubated with a recombinant anti-human leukocyte antigen class I (HLA-I) B8-PE IgG antibody (Miltenyi Biotec, Germany) (dilution: 1:50) for 30 min at room temperature. The expression profiles of HLA-I in groups were determined with a FACScan flow cytometer (BD, USA) using Cell Quest software. Fresh ECSs were used as controls.

### Apoptosis analyzed by the TUNEL method

Preserved ECSs were fixed in 4% paraformaldehyde solution for 30 min, and then 5 μm serial frozen sections were prepared. The TUNEL Apoptosis Detection Kit (Alexa Fluor 488) (YEASON, China) was used according to the manufacturer’s instructions. After straining with 2 μg/mL DAPI solution (Servicebio, China) at room temperature for 5 min, the slides were observed immediately under a fluorescence microscope. Green fluorescence was observed at 520 ± 20 nm with a standard fluorescent filter, and DAPI fluorescence was observed at 460 nm. Green fluorescent signals localized by Alexa Fluor 488–12-dUTP were only present in the apoptotic nuclei, while DAPI stained both apoptotic and nonapoptotic cells blue.

### Dihydroxyphenylacetic acid (DOPA) staining

To detect melanocyte viability in preserved ECSs, DOPA staining was performed. When exogenous levodopa is added and taken up, melanin particles were synthesized under the specific tyrosinase in melanocytes to make the cells appear black under the microscope [29]. The preserved ECS was reattached to a 60 mm dish and cultured for another 2 days. After fixation in 4% paraformaldehyde solution, fresh and preserved ECSs were used for this experiment. Following washing with PBS, the ECSs were incubated with 0.1% L-DOPA (Aladdin, China) (dissolved in PBS) at 37 °C for 5 h and counterstained with hematoxylin.

### Transplantation in nude mice

Twenty-four female C57BL/6 mice (six weeks old) were divided into four groups (*n* = 6 per group): 1) control group: mice with skin wounds and received no treatment; 2) fresh group: mice received fresh ECS transplantation; 3) refrigerated group: mice received ECSs stored in KGM supplemented with 0.5 mM ascorbic acid and 10 μM Y27632 at 4 degrees; and 4) frozen group: mice received ECSs cryopreserved in CP0 containing 0.1 M fructose, 0.5 mM ascorbic acid, and 10 μM Y27632. After the mice were anesthetized with 2% inhaled isoflurane, 10-mm-diameter full-thickness excisional skin wounds were created under sterile conditions. Then, the wounds were closed with transplantation of the ECSs by interrupted suturing. The mice were sacrificed at day 7 and day 14 postwounding. The wound tissues were harvested and then processed for analysis.

### Histological and immunohistochemical staining

Frozen sections or paraffin sections in groups were prepared as described above. Re-epithelialization and scar formation were assessed by H&E staining (Servicebio, China). Collagen deposition was detected by Masson’s trichrome staining (Servicebio, China). For immunohistochemical staining, the sections were blocked with 1% bovine serum albumin (Servicebio, China) and 0.5% Triton-X100 (Servicebio, China) and then separately treated with AE1/AE3 (Abcam, USA, prediluted), CD31(Abcam, USA, 1:500), CD68 (Abcam, USA, 1:500) and P63 (Abcam, USA, 1:500) mouse anti-human IgG antibody at 4 °C overnight. After washing three times with PBS for 5 min, the sections were incubated for 1 h with an HRP goat anti-mouse IgG antibody (Invitrogen, USA, 1:1000) at room temperature. Finally, the sections were stained with 3,3 N-diaminobenzidine tetrahydrochloride and counterstained with hematoxylin. The slides were covered with coverslips and examined under a microscope.

### Statistical analysis

Data analyses were performed using IBM SPSS Statistics 19.0 software (IBM Corporation, NY, USA). The data are presented as the mean ± standard deviation. Multiple comparisons were evaluated by one-way analysis of variance. A two-tailed t-test was adopted to evaluate the significance of the differences between two comparisons. The level of statistical significance was defined as *P* values < 0.05.

## Data Availability

Not applicable.

## Abbreviations

ECSs: Epithelial cell sheets
KGM: Keratinocyte growth medium
CPA: Cryoprotectant agents
DMSO: Dimethyl sulfoxide
HFKs: Human foreskin keratinocytes
H&E: Hematoxylin and eosin
DOPA: Dihydroxyphenylacetic acid
ECM: Extracellular matrix
SEM: Scanning electron microscopy
HLA-I: Human leukocyte antigen class I
TUNEL: TdT-mediated dUTP Nick-End Labeling
